# Phytochemicals, Two New Sulphur Glycosides and Two New Natural Products, from Shepherd’s Purse Seed and Their Activities

**DOI:** 10.3390/molecules29174145

**Published:** 2024-08-31

**Authors:** Zhen-Zhen Wei, Chun-Bo Ge, Yu-Jie Wang, Bin Li, Ying Tian, Ti-Qiang Zhou, Shu-Chen Liu, Jian-Feng Yi

**Affiliations:** 1 Integrated Chinese and Western Medicine Institute for Children Health and Drug Innovation, Institute of Chinese Medicine, Institute for Advanced Study, Jiangxi University of Chinese Medicine, Nanchang 330004, China; qilerongrong11@126.com (Z.-Z.W.); wangyujie4213@163.com (Y.-J.W.); 2 College of Life Sciences, Hebei University, Baoding, 071002, China; gechunb@163.com; 3 Department of Pharmaceutical Science, Beijing Institute of Radiation Medicine, Beijing 100850, China; jkylibin@hotmail.com (B.L.); hq6106@aliyun.com (Y.T.); 4 Advanced Research Institute of Multidisciplinary Science, School of Life Science, Beijing Institute of Technology, Beijing 100081, China

**Keywords:** *Capsella bursa-pastoris*, organosulfur compound, cruciferae, sulphur glycosides, natural product

## Abstract

Two new sulfur glycosides, bursapastoris A–B (**3**–**4**), were extracted and isolated from shepherd’s purse seed, along with two new natural products, 11-(methylsulfinyl)undecanoic acid (**2**) and 10-(methylsulfinyl)decanoic acid (**1**). Their structures were determined though infrared spectroscopy, one-dimensional nuclear magnetic resonance (^1^H and ^13^C), and electrospray ionization mass spectrometry. Additionally, the structures of **3**–**4** were further identified by two-dimensional nuclear magnetic resonance (HMBC, HSQC, ^1^H-^1^H COSY, and NOESY). Compounds **1**–**4** showed relatively favorable docking to NF-*κ*B. Unfortunately, we only discovered that compound **1**–**4** had weak anti-radiation activity at present. Therefore, further research regarding the biological activity of these organosulfur compounds is required at a later stage.

## 1. Introduction

*Capsella bursa*-*pastoris* (shepherd’s purse), which belongs to the cruciferae family, is distributed all over the world and is used as a vegetable as well as a folk medicine [1]. Shepherd’s purse has been used as a remedy for centuries and has garnered attention for its various medicinal properties, including antihemorrhoidal [2], anti-inflammatory [3], antimicrobial [4], and hemostatic [5,6] activities, among others. In traditional Chinese medicine, it is used for dysentery and eye disorders, with people chewing the seeds of shepherd’s purse to improve vision. The seeds not only have high nutritional value but also possess multifarious medicinal benefits, such as improving visual acuity, alleviating ocular discomfort, tonifying the digestive system, and enhancing vision.

Organosulfur compounds play a crucial role as therapeutic agents in medicinal chemistry, holding the key to the health-promotion benefits as they possess biological activities, including antihypertensive [7], antiatherosclerotic [8], antifungal [9], antibacterial [10], and anticancer effects [11], among others. Sulfur exhibits unique chemical properties, including a wide range of oxidation states and versatility in reactions that facilitate essential biological processes and redox biochemistry [12]. The presence of sulfur is responsible for the distinctive characteristics of organosulfur compounds, which have been harnessed in the treatment of diseases mediated by oxidative stress. These compounds are mainly present in fruits, vegetables, and edible mushrooms, and undergo slow biotransformation into various metabolites such as thiols, sulfides, and peptides during biosynthesis [13]. However, the distinctive biological activity of these metabolites remains largely unexplored. As such, there is a need for further development of organic sulfur species. 

**Figure 1:**
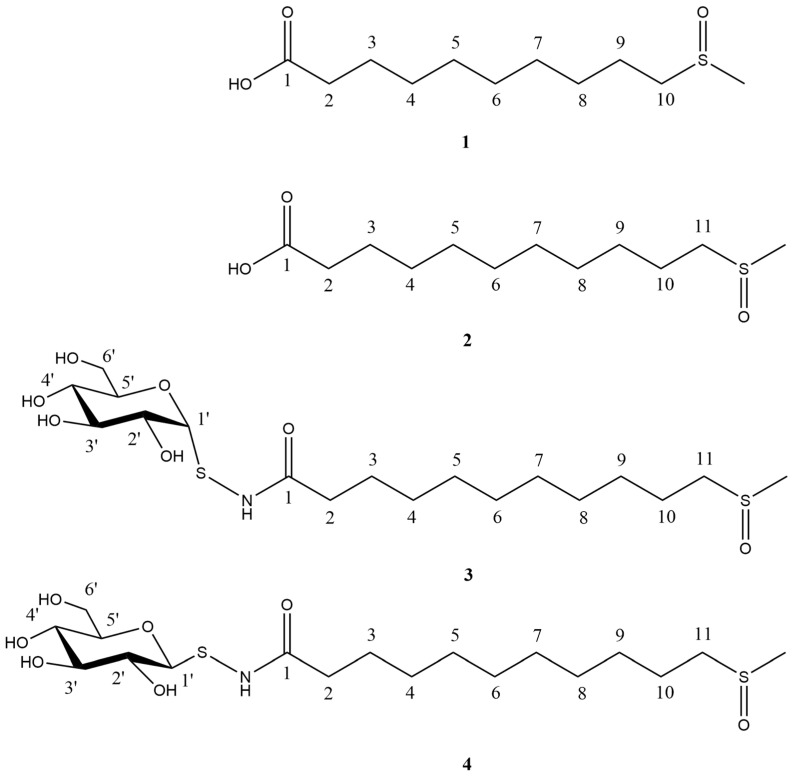
Chemical structures of compounds **1**–**4**.

Previously, we identified four organosulfur compounds in shepherd’s purse seeds [14]. Our current research is focused on the exploration of additional organosulfur compounds present in the shepherd’s purse seed, which has prompted us to continue conducting a comprehensive phytochemical investigation on these seeds [15]. In this study, we have discovered four more organosulfur compounds, including two new sulfur glycosides (**3**–**4**) and two new natural products (**1**–**2**) (Figure 1). Then, the molecular docking method was used to screen the compounds, and it was found that compounds **1**–**4** showed relatively favorable docking to NF-*κ*B.

## 2. Results

### 2.1. Structure Elucidation

Compound **1** had the chemical formula of C_11_H_22_O_3_S as its MS *m*/*z* 233.1207 [M-H]^−^ (*calcd.* for C_11_H_21_O_3_S^−^, 233.1211). Its ^1^H NMR spectrum (Figure S1 and Table S1) showed nine methylene proton signals at *δ*_H_ 2.72, 2.63, 2.19, 1.61, 1.48, 1.38, and 1.25–1.30, and a methyl group at *δ*_H_ 2.51. Its ^13^C NMR spectrum (Figure S2 and Table S1) exhibited seven methylene carbon signals at *δ*_C_ 28.7 (double), 28.6 (double), 28.2, 24.5, and 22.0, a methyl carbon signal (38.0, CH_3_SO), two characteristic methylene carbon signals (53.2, 33.7), and one carbonyl carbon signal (174.6). Based on the aforementioned findings and in comparison with the database, it has been determined to be 10-methylsulfinyl-decanoic acid.

Compound **2** possessed the chemical formula of C_11_H_22_O_3_S as its MS *m*/*z* 247.1427 [M-H]^−^ (*calcd.* for C_12_H_23_O_3_S^−^, 247.1368) and 495.2874 [2M-H]^−^ (*calcd.* for C_24_H_47_O_6_S_2_^−^, 495.2814) (Figure S6). Its NMR data (Figures S4 and S5, Table S1) were similar to those of **1** but with an additional methylene fragment. As a result, it was confirmed as 11-methylsulfinyl-undecanoic acid.

Compound **3** had the chemical formula of C_18_H_35_NO_7_S_2_ according to its MS *m*/*z* 476.1549 [M+Cl]^−^ (calcd for C_18_H_35_NO_7_S_2_Cl^−^, 476.1543). Its IR spectrum revealed the presence of N-H (overlapped 3431, 866, 668 cm^−1^), OH (overlapped 3431, 1095, 1055, 1010 cm^−1^), CH_2_ (2922, 2852, 1466 cm^−1^), and C=O (1686 cm^−1^). Its ^1^H NMR spectrum (Table 1 and Figure S7) revealed ten methylene proton signals at *δ*_H_ 2.80, 2.29, 1.75, 1.61, 1.48, and 1.32–1.38, and a methyl group (2.63, 3H). In addition, it also showed the characteristic anomeric proton signal (5.43, 1H, d, *J* = 5.8 Hz, H-1′) and other proton signals of glycone at *δ*_H_ 4.07, 3.78, 3.75, 3.74, 3.49, and 3.37. Its ^13^C NMR spectrum (Figure S8 and Table 1) showed eighteen signals, including eight methylene carbon signals at *δ*_C_ 30.4 (double), 30.3 (C-6), 30.2 (double), 29.7, 26.9, and 23.6, one methyl carbon signal (38.1, CH_3_SO), two methylene carbon signals at *δ*_C_ 54.9 and 37.3, one six-carbon sugar carbon signal at *δ*_C_ 90.3, 75.8, 74.6, 73.1, 71.2, and 62.4, and a carbonyl carbon signal (179.2). Its HSQC united with the ^13^C NMR spectrum revealed that the glucose fragment exhibited an anomeric carbon signal at *δ*_C_ 90.3, indicating its connection with a sulfur atom [16].

**Figure 2:**
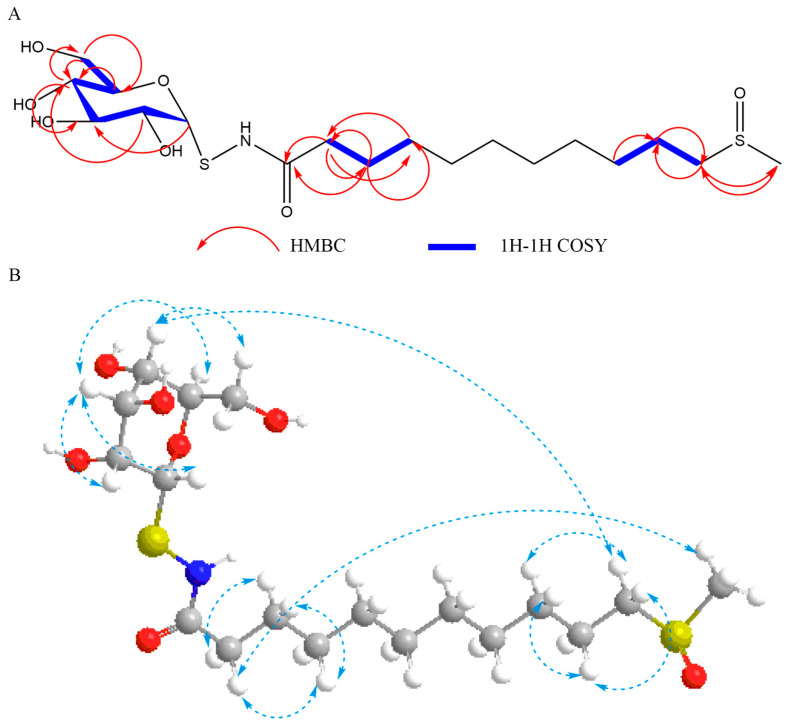
^1^H-^1^H COSY, HMBC, and NOESY correlations of bursapastoris A. (**A**) ^1^H-^1^H COSY and HMBC correlations; (**B**) NOESY correlation (light blue dashed arrow), white: hydrogen; grey: carbon; red: oxygen; yellow: sulfur.

The glucose was obtained and analyzed through acid hydrolysis and HPLC analysis of **3**. Meanwhile, the negative optical rotation and the small coupling constant (5.8 Hz) of the anomeric proton at *δ*_H_ 5.43 (H-1′) implies that the glucose is in the *α*-*D*-configuration [17]. Eventually, the assignment of protons and carbons were reached by the HMBC, HSQC, and ^1^H-^1^H COSY spectra (Figure 2A). The nuclear overhauser effect (NOE) correlations were observed (Figure 2B). Therefore, its structure was elucidated (Figure 1) and designated as bursapastoris A.

Compound **4** had the chemical formula of C_18_H_35_NO_7_S_2_ as quasi-molecular ion peaks 440.1751 [M-H]^−^ (calcd for C_18_H_34_NO_7_S_2_^−^, 440.1777) and 476.1546 [M+Cl]^−^ (calcd for C_18_H_35_NO_7_S_2_Cl^−^, 476.1543). Its IR spectrum revealed the presence of N-H (3267, 891, 561 cm^−1^), OH (3431, 1090, 1051, 1007 cm^−1^), CH_2_ (2923, 2853, 1468 cm^−1^), and C=O (1651 cm^−1^). Its NMR spectra (Table 1, Figures S16 and S17) closely resembled those of compound **3**, except for the distinctive anomeric proton signal (4.12, 1H, d, 9.36 Hz, H-1′). Acid hydrolysis combined with HPLC analysis confirmed the presence of glucose in compound **4**. In addition, the negative optical rotation and the large coupling constant (9.36 Hz) of the anomeric proton at *δ*_H_ 4.12 (H-1′) indicate that the glucose is in the *β*-*D*-configuration [16].

**Figure 3:**
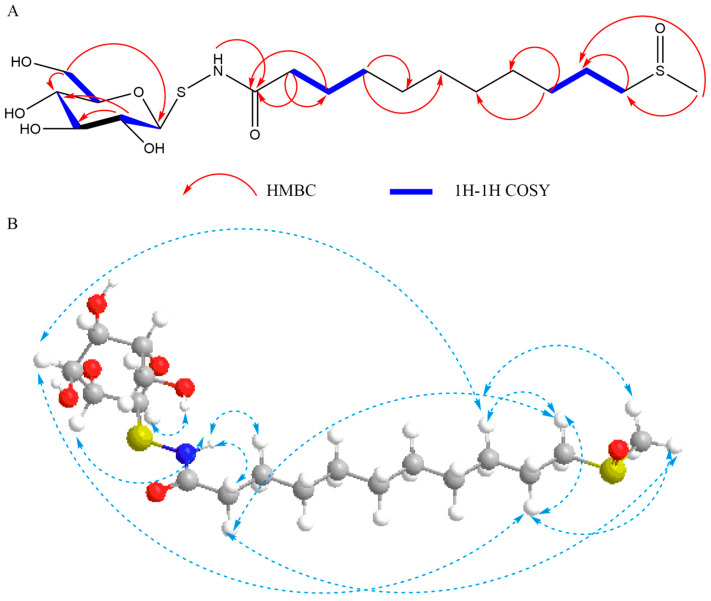
^1^H-^1^H COSY, HMBC, and NOESY correlations of bursapastoris B. (**A**) ^1^H-^1^H COSY and HMBC correlations; (**B**) NOESY correlation (light blue dashed arrow), white: hydrogen; grey: carbon; red: oxygen; yellow: sulfur.

The assignment of carbons and protons were ultimately achieved through the analysis of HMBC, HSQC, and ^1^H-^1^H COSY spectra (Figure 3A). The nuclear overhauser effect (NOE) correlations were observed in the NOESY experiment (Figure 3B). The structure of **4** was elucidated (Figure 1) and designated as bursapastoris B based on the aforementioned evidence.

### 2.2. Structural Information of **1**–**2**

Compound **1**. White amorphous powder, ^1^H and ^13^C NMR (DMSO-*d*_6_), see Table S1. HR-ESI-MS (negative-ion mode) *m*/*z*: 233.1207 [M-H]^−^.

Compound **2**. White amorphous powder, ^1^H and ^13^C NMR (CD_3_OD-*d*_4_), see Table S1; HR-ESI-MS *m*/*z*: 247.1427 [M-H]^−^, 495.2874 [2M-H]^−^.

Compound **3**. White amorphous powder, ^1^H and ^13^C NMR (CD_3_OD-*d*_6_), see Table 1. HR-ESI-MS *m*/*z*: 476.1549 [M+Cl]^−^. IR (KBr) *ν*_max_: N-H (overlapped 3431, 866, 668 cm^−1^), C=O (1686 cm^−1^), CH_2_ (2922, 2852, 1466 cm^−1^), and OH (overlapped 3431, 1095, 1055, 1010 cm^−1^).

Compound **4**. White amorphous powder, ^1^H and ^13^C NMR (DMSO-*d*_6_), see Table 1. HR-ESI-MS (negative-ion) *m*/*z*: 440.1751 [M-H]^−^, 476.1546 [M+Cl]^−^. IR (KBr) *ν*_max_: N-H (3267, 891, 561 cm^−1^), C=O (1651 cm^−1^), CH_2_ (2923, 2853, 1468 cm^−1^), and OH (3431, 1090, 1051, 1007 cm^−1^).

### 2.3. Proposed Biosynthetic Pathway

**Figure 4:**
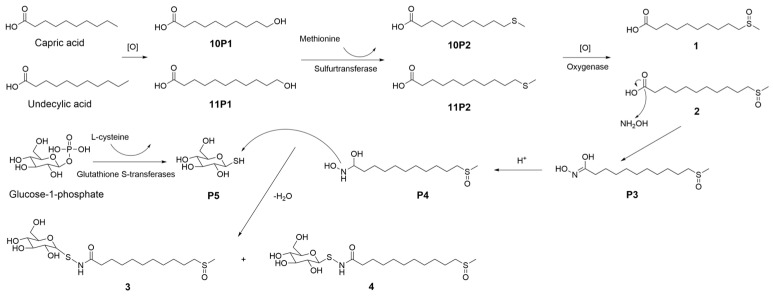
Proposed biosynthetic pathway for compounds **1**–**4**.

A plausible biogenetic pathway [18] was proposed for the formation of these organosulfur compounds, involving the following key steps (Figure 4): (1) an oxidation reaction converted fatty acids to 10P1 and 11P1; (2) the enzymatic catalytic reaction of 10P1 or 11P1 and methionine to 10P2 or 11P2 was catalyzed by sulfurtransferase; (3) the thioether was easily oxidized into compounds **1** and **2** by oxygenase; (4) P3 was derived from compound **2** through a reaction with a hydroxyamine unit; (5) protonation of P3 leads to a proposed intermediate P4; (6) P5 was derived from glucose-1-phosphate through a reaction with an L-cysteine unit; (7) compounds **3** and **4** were derived from the combination of P4 with P5 by the leaving of H_2_O.

### 2.4. Anti-Radiation Activity

**Figure 5:**
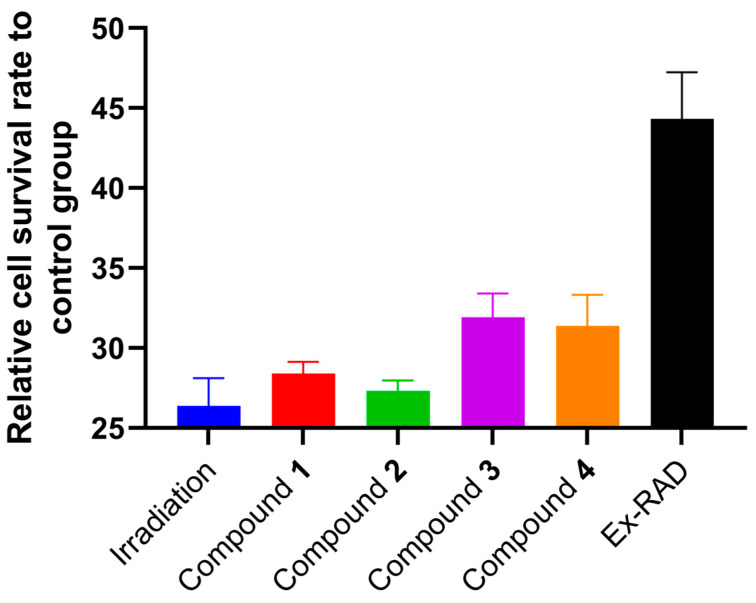
The anti-radiation activities on X-ray irradiated AHH-1 cells of compounds **1**–**4**.

In the investigation of the activity of compounds **1**–**4**, it was observed that they did not exhibit any significant anti-inflammatory, anti-tumor, or anti-oxidation properties. However, organosulfur compounds have been known to demonstrate promising effects in protecting against radiation damage, such as diallyl sulfide [19], cysteamine [20], amifostine [21,22], and others. Therefore, it is intriguing to explore whether compounds **1**–**4** (four natural organic sulfur compounds) possess potential for mitigating radiation-induced damage. The anti-radiation activities of compounds **1**–**4** were evaluated using X-ray irradiated AHH-1 cells as a model, and the result showed that the four organosulfur compounds had weak radioprotective effects (Figure 5).

### 2.5. Molecular Docking Analysis

Although there was no direct evidence of biological activity for these compounds from the experiment, we sought to investigate potential clues through molecular docking. This is based on the consideration that these four organosulfur compounds may possess some level of biological activity. The nuclear transcription factor Nuclear Factor kappa-B (NF-*κ*B) plays a crucial role in regulating the expression of numerous genes [23]. NF-*κ*B is considered to be a component of the stress response, as it can be induced by various stimuli including UV radiation, pharmacological agents, and stressful conditions [24,25,26]. The docking energies of compounds **1**–**4** with NF-*κ*B are −4.76, −4.20, −3.28, and −4.04 KCal/mol, respectively. These values indicate that compounds **1**–**4** exhibited relatively favorable docking to NF-*κ*B.

**Figure 6:**
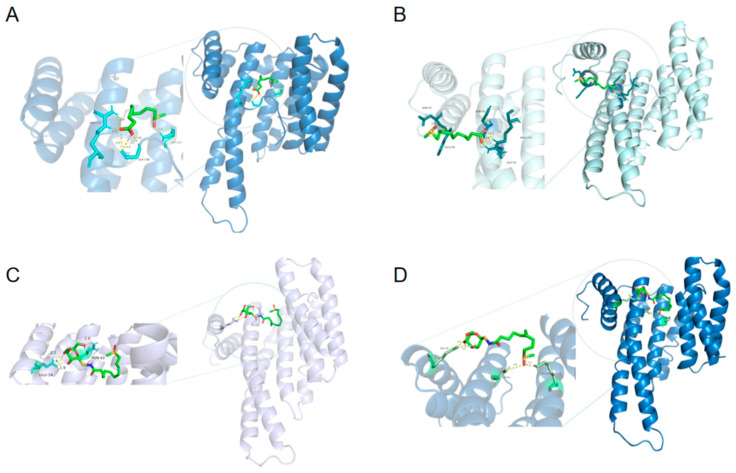
Lowest-energy docked pose of compounds with NF-*κ*B. (**A**) **1**; (**B**) **2**; (**C**) **3**; (**D**) **4**.

The binding site of NF-*κ*B with compounds **1**–**4** includes hydrophobic pockets surrounded by GLY-130, LYS-122, ARG-132, GLY-53, GLU-20, ASP-21, ASN-50, ARG-131, GLU-14, ASN-42, GLU-14, SER-45, and LYS-122 (Figure 6). Take compound **3** as an example: the docked pose of compound **3** with NF-*κ*B, exhibiting the lowest energy, demonstrates significant interactions with GLU-14 and ASN-42. The docked compound **3** is near to GLU-14 with closest distance 1.9 Å. Although compounds **1**–**4** have exhibited highly favorable docking scores with NF-*κ*B, it is important to note that NF-κB is associated with a wide range of biological activities. The specific biological activities of compounds **1**–**4** in relation to NF-*κ*B are not yet clear, and further research is required to explore the potential biological activity of these four organosulfur compounds in future experiments.

## 3. Materials and Methods

### 3.1. General Experimental Procedures

The experimental procedures were carried out following standard protocols, and NMR experiments were conducted using an AVANCE NEO 600 instrument (Bruker BioSpin, Billerica, MA, USA). The HR-ESI-MS analysis was conducted using an TOF LC/MS system (Agilent Technologies Co., Ltd., Santa Clara, CA, USA). The preparative HPLC separation was conducted using an LC-3000 liquid chromatography system (Beijing Chuangxin Hengtong Technology Co., Ltd., Beijing, China). The column utilized was an Innoval C_18_ column (21.2 × 250 nm, 5 μm, Tianjin Bona Aijieer Technology Co., Ltd., Tianjin, China). The IR spectra were analyzed using a Frontier FT-IR instrument with KBr pellets. The column chromatography was conducted utilizing silica gel (200–300 mesh, Marine Chemical Factory, Qingdao, Shandong, China), Sephadex LH-20 (Pharmacia, Stockholm, Sweden), and Macroporous resin AB-8 (Nan Kai College Chemical Inc., Tianjin, China). TLC was performed on GF254 plates pre-coated with silica gel 60 (5–20 μm, Yantai Huayang New Material Technology Co., Ltd., Yantai, Shandong, China).

### 3.2. Plant Materials

The seeds of *Capsella bursa*-*pastoris* were obtained from the Bozhou Traditional Chinese Medicine Trading Center, and their producing area is located at a geographical coordinate of 114°52′–115°31′ E, 32°35′–33°08′ N, with an approximate altitude of 40 m in Anhui province, China, in October 2021. The identification was performed by Professor Bin Li from the Department of Pharmaceutical Chemistry at Beijing Institute of Radiation Medicine. The voucher specimen (No. 2021-1006) has been deposited in the specimen cabinet at the Beijing Institute of Radiation Medicine.

### 3.3. Isolations of Compounds

The ethanol extraction process was used to obtain a concentrated residue of 6.69 kg from 80 kg of dried Capsella bursa-pastoris seeds, using 70% ethanol as the solvent. Then, the extract was dissolved in water and underwent liquid-liquid extraction using petroleum ether (PE), dichloromethane, ethyl acetate (EtOAc), and *n*-butanol (*n*-BuOH). The *n*-BuOH extract (1000 g) underwent fractionation into four fractions (25%, 50%, 70%, and 95% ethanol) utilizing the separation technique of macroreticular resin attraction (AB-8) [27,28]. The 25% ethanol fraction was subjected to purification using various techniques, including column chromatography on silica gel, Sephadex LH-20 gel, and preparative HPLC. This process successfully led to the isolation of bursapastoris A (32.1 mg), bursapastoris B (30.4 mg), 10-(methylsulfinyl) decanoic acid (207.4 mg), and 11-(methylsulfinyl) undecanoic acid (587.2 mg).

### 3.4. Cells and Cell Culture

AHH-1 cells were cultured in Dulbecco’s Modified Eagle’s Medium (Macgene, Beijing, China) supplemented with 10% (*v*/*v*) fetal bovine serum (Gibco, Carlsbad, CA, USA), 100 U/mL penicillin (Macgene, Beijing, China), and 100 μg/mL Streptomycin sulfate (Macgene, Beijing, China) at a temperature of 37 °C under a CO_2_ concentration of 5%.

### 3.5. Anti-Radiation Assay

To investigate the radiation protection effects of compounds **1**–**4** against apoptosis induced by X-ray radiation in the AHH-1cells [29], AHH-1 cells were seeded in 96-well plates at a density of 2.6 × 10^4^ cells per well and incubated overnight at 37 °C under 5% CO_2_. Then, the cells were treated with compounds **1**–**4** at a concentration of 100 μM for 2 h followed by radiation. For the radiation, cells preincubated with compounds **1**–**4** were exposed to X-ray beams with a dose of 8 Gy in total. The cells with radiant inducement were used as an irradiation group, and recilisib (Ex-RAD), a radioprotectant, was used as a positive control group at a concentration of 100 μM. After incubating under the same conditions for 24 h, cell viability was monitored by CCK8, and the absorbance of each well at 450 nm was measured on a microplated reader (Multiskan MK-3, Thermo, Waltham, MA, USA).

### 3.6. Molecular Docking Assay

The docking study was conducted on pertinent protein targets associated with inflammation utilizing the AutoDock 4.2.6 and AutoDockTools 1.5.7 program [30].

## 4. Conclusions

In summary, we have isolated, purified, and identified two new sulfur glycosides and two new natural products from shepherd’s purse seed. The structures were elucidated through the utilization of NMR experiments as well as mass spectrometry. Despite receiving less research attention in the past, the seeds of shepherd’s purse contain natural organic sulfur compounds that warrant further exploration. We are confident that additional new organic sulfur compounds can be obtained from it to enrich the types of organosulfur compounds and sulfur glycosides in plants. Molecular docking studies have shown that compounds **1**–**4** exhibited relatively favorable docking to NF-*κ*B; however, these four organosulfur compounds demonstrated weak radioprotective effects. This may be attributed to the fact that their outstanding biological activity has not been identified, thus necessitating further screening and verification through additional experiments in our later stages.

## Figures and Tables

**Table 1:** NMR data of compounds **3** and **4** (600 MHz for ^1^H, 150 MHz for ^13^C).

No	Compond 3 (CD_3_OD)		Compond 4 (DMSO-*d*_6_)	
	*δ*_H_ (*J* in Hz)	*δc*	*δ*_H_ (*J* in Hz)	*δ*c
1	-	179.2	-	177.7
2	2.29 (2H, t, 7.3)	37.3	2.27 (2H, m)	35.2
3	1.61 (2H, m)	26.9	1.51 (2H, m)	25.2
4	1.32–1.38 (2H, m)	29.7	1.24–1.30 (2H, m)	28.4
5	1.32–1.38 (2H, m)	30.2	1.24–1.30 (2H, m)	28.6
6	1.32–1.38 (2H, m)	30.3	1.24–1.30 (2H, m)	28.8
7	1.32–1.38 (2H, m)	30.4	1.24–1.30 (2H, m)	28.7
8	1.32–1.38 (2H, m)	30.4	1.24–1.30 (2H, m)	28.4
9	1.48 (2H, m)	30.2	1.37 (2H, m)	28.1
10	1.75 (2H, m)	23.6	1.61 (2H, m)	21.9
11	2.80 (2H, m)	54.9	2.73 (1H, m) 2.62 (1H, m)	53.2
CH_3_SO	2.63 (3H, s)	38.1	2.50 (3H, s)	38.0
1′	5.43 (1H, d, 5.8)	90.3	4.12 (1H, d, 9.4)	88.9
2′	3.75 (1H, dd, 5.8, 3.9)	73.1	2.99 (1H, m)	70.2
3′	4.07 (1H, m)	74.6	2.86 (1H, m)	70.1
4′	3.37 (1H, t, 9.9)	71.2	3.19 (1H, m)	76.6
5′	3.49 (1H, t, 9.3)	75.8	3.24 (1H, m)	81.5
6′	3.78 (1H, dd, 11.8, 2.5) 3.74 (1H, dd, 11.0, 5.1)	62.4	3.69 (1H, dd, 11.8, 5.4) 3.41 (1H, m)	61.3

Note: s (a single peak), d (a double peak), t (a triple peak), q (a quadruple peak), dd (doublet of doublets) and m (a multiple peak).

## Data Availability

All other datasets generated for this study are included in the article/Supplementary Material.

## Supplementary Materials

The following supporting information can be downloaded at: https://www.mdpi.com/article/10.3390/molecules29174145/s1, Figure S1: ^1^H-NMR spectrum of **1**; Figure S2: ^13^C-NMR spectrum of **1**; Figure S3: HR-ESI-MS spectrum of **1**; Figure S4: ^1^H-NMR spectrum of **2**; Figure S5: ^13^C-NMR spectrum of **2**; Figure S6: HR-ESI-MS spectrum of **2**; Figure S7: ^1^H-NMR spectrum of **3**; Figure S8: ^13^C-NMR spectrum of **3**; Figure S9: IR spectrum of **3**; Figure S10: DEPT spectrum of **3**; Figure S11: HSQC spectrum of **3**; Figure S12: HMBC spectrum of **3**; Figure S13: ^1^H-^1^H COSY spectrum of **3**; Figure S14: NOESY spectrum of **3**; Figure S15: HR-ESI-MS spectrum of **3**; Figure S16: ^1^H-NMR spectrum of **4**; Figure S17: ^13^C-NMR spectrum of **4**; Figure S18: IR spectrum of **4**; Figure S19: DEPT spectrum of **4**; Figure S20: HSQC spectrum of **4**; Figure S21: HMBC spectrum of **4**; Figure S22: ^1^H-^1^H COSY spectrum of **4**; Figure S23: NOESY spectrum of **4**; Figure S24: HR-ESI-MS spectrum of **4**; Table S1: NMR data of **1** and **2**.

## References

[1] Peng J., Hu T., Li J., Du J., Zhu K., Cheng B., Li K. (2019). Shepherd’s Purse Polyphenols Exert Its Anti-Inflammatory and Antioxidative Effects Associated with Suppressing MAPK and NF-κB Pathways and Heme Oxygenase-1 Activation. Oxid. Med. Cell Longev..

[2] Apaydin Yildirim B., Aydin T., Kordali S., Yildirim S., Cakir A., Yildirim F. (2020). Antihemorrhoidal activity of organic acids of *Capsella bursa-pastoris* on croton oil-induced hemorrhoid in rats. J. Food Biochem..

[3] Cha J.M., Suh W.S., Lee T.H., Subedi L., Kim S.Y., Lee K.R. (2017). Phenolic Glycosides from *Capsella bursa-pastoris* (L.) Medik and Their Anti-Inflammatory Activity. Molecules.

[4] Wątły J., Szarszoń K., Mikołajczyk A., Grelich-Mucha M., Matera-Witkiewicz A., Olesiak-Bańska J., Rowińska-Żyrek M. (2023). Zn(II) Induces Fibril Formation and Antifungal Activity in Shepherin I, An Antimicrobial Peptide from *Capsella bursa-pastoris*. Inorg. Chem..

[5] Öztürk O.U., Ugur M., Güzel Y., Öztürk M.A., Gürsoy D., Doğan S., Temiz M. (2022). Hemostatic effects of traditional *Inula viscosa* and *Capsella bursa-pastoris* plant mixture extract on rat liver parenchymal bleeding model. Ulus. Travma. Acil. Cerrahi. Derg..

[6] Naafe M., Kariman N., Keshavarz Z., Khademi N., Mojab F., Mohammadbeigi A. (2018). Effect of Hydroalcoholic Extracts of *Capsella bursa-pastoris* on Heavy Menstrual Bleeding: A Randomized Clinical Trial. J. Altern. Complement Med..

[7] Schepetkin I.A., Kirpotina L.N., Khlebnikov A.I., Balasubramanian N., Quinn M.T. (2019). Neutrophil Immunomodulatory Activity of Natural Organosulfur Compounds. Molecules.

[8] Torres Palazzolo C., Martín Giménez V.M., Mazzei L., De Paola M., Quesada I., Cuello Carrión F.D., Fornés M.W., Camargo A.B., Castro C., Manucha W. (2022). Consumption of oil macerated with garlic produces renovascular protective effects in adult apolipoprotein E-deficient mice. Food Funct..

[9] Sorlozano-Puerto A., Albertuz-Crespo M., Lopez-Machado I., Gil-Martinez L., Ariza-Romero J.J., Maroto-Tello A., Baños-Arjona A., Gutierrez-Fernandez J. (2020). Antibacterial and Antifungal Activity of Propyl-Propane-Thiosulfinate and Propyl-Propane-Thiosulfonate, Two Organosulfur Compounds from *Allium cepa*: In Vitro Antimicrobial Effect via the Gas Phase. Pharmaceuticals.

[10] Bhatwalkar S.B., Mondal R., Krishna S.B.N., Adam J.K., Govender P., Anupam R. (2021). Antibacterial Properties of Organosulfur Compounds of Garlic (*Allium sativum*). Front. Microbiol..

[11] Ahmed T., Wang C.K. (2021). Black Garlic and Its Bioactive Compounds on Human Health Diseases: A Review. Molecules.

[12] Egbujor M.C., Petrosino M., Zuhra K., Saso L. (2022). The Role of Organosulfur Compounds as Nrf2 Activators and Their Antioxidant Effects. Antioxidants.

[13] Lu Y., Zhang M., Huang D. (2022). Dietary Organosulfur-Containing Compounds and Their Health-Promotion Mechanisms. Annu Rev. Food Sci. Technol..

[14] Wei Z.Z., Zhou T.Q., Xia Z.M., Liu S.F., Li M., Zhang G.J., Tian Y., Li B., Wang L. (2022). Four organosulfur compounds from the seeds of *Capsella bursa-pastoris* and their anti-inflammatory activities. Nat. Prod. Res..

[15] Zhou T.Q., Wei Z.Z., Zhang J.R., Dong J.H., Liu C.Y., Jiang C.Z., Xia Z.M., Liu S.F., Li M., Zhang G.J. (2023). Phytochemical Constituents from the Seeds of *Capsella bursa-pastoris* and Their Antioxidant Activities. Plant Foods Hum. Nutr..

[16] Feng W.S., Li C.G., Zheng X.K., Li L.L., Chen W.J., Zhang Y.L., Cao Y.G., Gong J.H., Kuang H.X. (2016). Three new sulphur glycosides from the seeds of *Descurainia sophia*. Nat. Prod. Res..

[17] Wang S., Shi P., Qu L., Ruan J., Yang S., Yu H., Zhang Y., Wang T. (2017). Bioactive Constituents Obtained from the Seeds of *Lepidium apetalum* Willd. Molecules.

[18] Petkowski J.J., Bains W., Seager S. (2018). Natural Products Containing a Nitrogen-Sulfur Bond. J. Nat. Prod..

[19] Katoch O., Khan G.A., Dwarakanath B.S., Agrawala P.K. (2012). Mitigation of hematopoietic radiation injury by diallyl sulphide. J. Environ. Pathol. Toxicol. Oncol..

[20] Brucer M., Mewissen D.J. (1957). Late effects of gamma radiation on mice protected with cysteamine or cystamine. Nature.

[21] Ji L., Cui P., Zhou S., Qiu L., Huang H., Wang C., Wang J. (2023). Advances of Amifostine in Radiation Protection: Administration and Delivery. Mol. Pharm..

[22] Kouloulias V.E., Kouvaris J.R. (2008). Cytoprotective efficacy of amifostine against radiation-induced rectal toxicity: Objective and subjective grading scales for radiomucositis. Molecules.

[23] Poma P. (2020). NF-*κ*B and Disease. Int. J. Mol. Sci..

[24] Hayden M.S., Ghosh S. (2008). Shared principles in NF-kappaB signaling. Cell.

[25] Lawrence T. (2009). The nuclear factor NF-kappaB pathway in inflammation. Cold Spring Harb. Perspect. Biol..

[26] Oeckinghaus A., Hayden M.S., Ghosh S. (2011). Crosstalk in NF-*κ*B signaling pathways. Nat. Immunol..

[27] Wei Z.Z., Zhou T.Q., Zhang J.R., Xia Z.M., Liu S.F., Liu C.Y., Li M., Zhang G.J., Tian Y., Li B. (2023). Discovery of pentacyclic triterpenoid glycosides with anti-proliferative activities from *Ardisialindleyana*. Carbohydr. Res..

[28] Zhou T.Q., Wei Z.Z., Fu Q.Y., Rui Q., Zhang G.J., Li B., Dong J.X., Zeng C.C. (2023). A new oleanane-type triterpene from *Ardisia lindleyana* D.Dietr and its cytotoxic activity. Nat. Prod. Res..

[29] Yang J., Zhou Y., Liu H., Wang J., Hu J. (2015). MCI extraction from Turkish galls played protective roles against X-ray-induced damage in AHH-1 cells. Int. J. Clin. Exp. Pathol..

[30] El-Hachem N., Haibe-Kains B., Khalil A., Kobeissy F.H., Nemer G. (2017). AutoDock and AutoDockTools for Protein-Ligand Docking: Beta-Site Amyloid Precursor Protein Cleaving Enzyme 1(BACE1) as a Case Study. Methods Mol. Biol..

