# Supplementary material for: Phytochemicals, Two New Sulphur Glycosides and Two New Natural Products, from Shepherd’s Purse Seed and Their Activities
Source: Molecules. 2024 Aug 31;29(17):4145. doi: 10.3390/molecules29174145 (PMC11397210; doi:10.3390/molecules29174145)
Supplement: Supplementary file 1 [file molecules-29-04145-s001.zip › molecules-3167136-supplementary.pdf]

Supplementary Material

Phytochemicals, Two New Sulphur Glycosides and Two New Natural Products, from Shepherd's Purse Seed and Their Activities

Zhen-Zhen Wei [1,†], Chun-Bo Ge [2,†], Yu-Jie Wang [1], Bin Li [2], Ying Tian [2], Ti-Qiang Zhou [3,*], Shu-Chen Liu [2,*] and Jian-Feng Yi [1,*]

[1] Integrated Chinese and Western Medicine Institute for Children Health and Drug Innovation, Institute of Chinese Medicine, Institute for Advanced Study, Jiangxi University of Chinese Medicine, Nanchang 330004, China; qilerongrong11@126.com (Z.-Z.W.); wangyujie4213@163.com (Y.-J.W.)

[2] Department of Pharmaceutical Science, Beijing Institute of Radiation Medicine, Beijing 100850, China; gechunb@163.com (C.-B.G.); jkylibin@hotmail.com (B.L.); hq6106@aliyun.com (Y.T.)

[3] Advanced Research Institute of Multidisciplinary Science, School of Life Science, Beijing Institute of Technology, Beijing 100081, China

* Correspondence: zhoutiqiang@163.com (T.-Q.Z.); lsc_biorm2019@163.com (S.-C.L.); jianfeng_yi21@163.com (J.-F.Y.)

† These authors contributed equally to this manuscript.

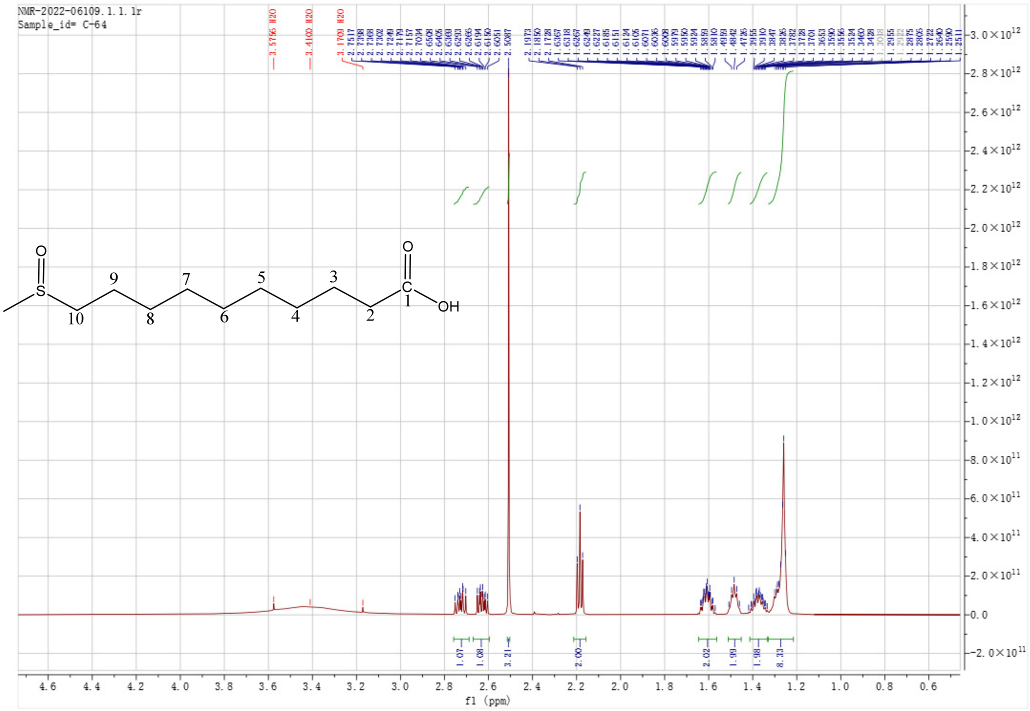

Supplementary Figure S1. ^{1}H-NMR spectrum of **1**

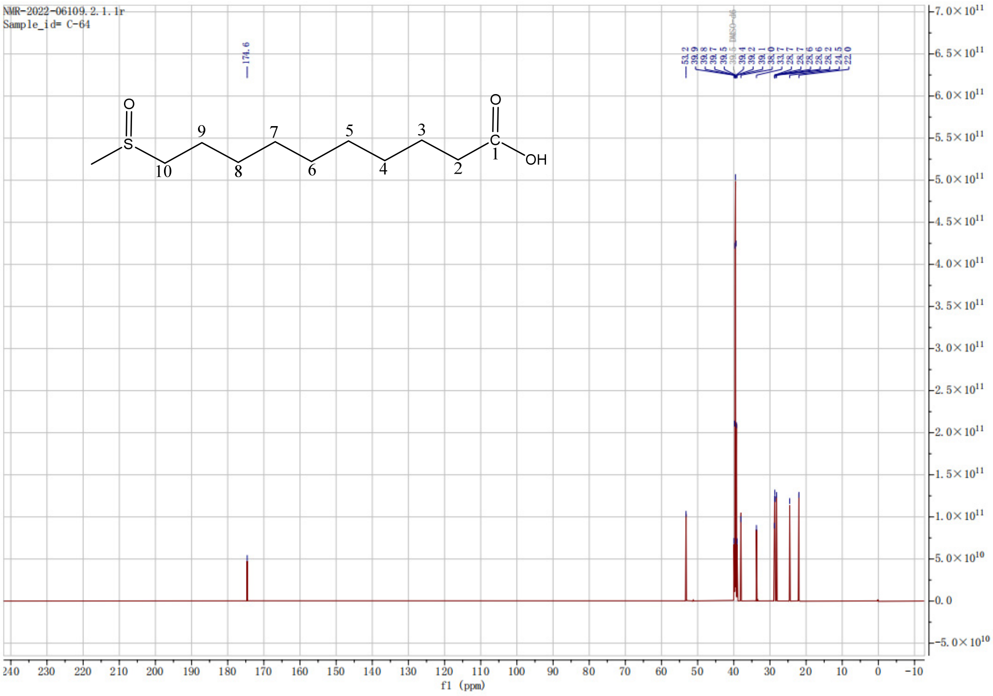

Supplementary Figure S2. ^{13}C-NMR spectrum of **1**

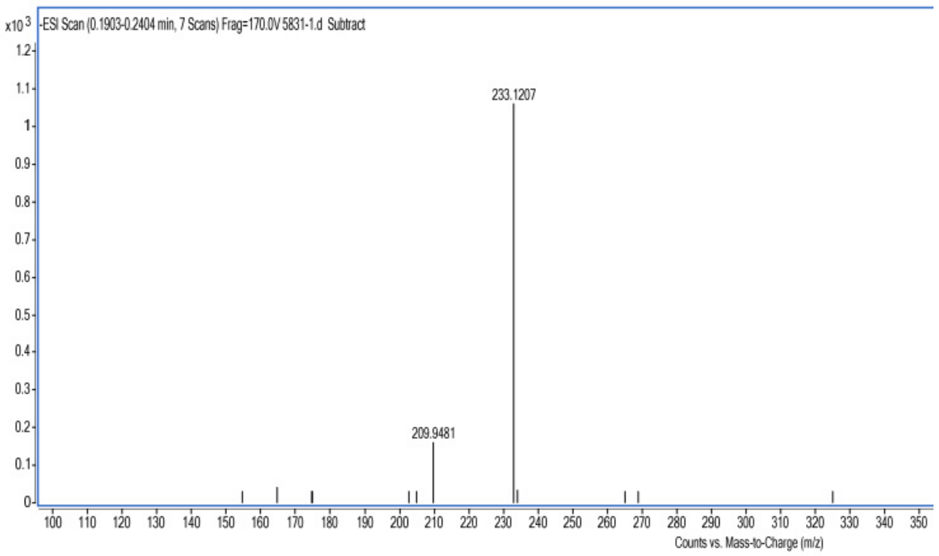

Supplementary Figure S3. HR-ESI-MS spectrum of **1**

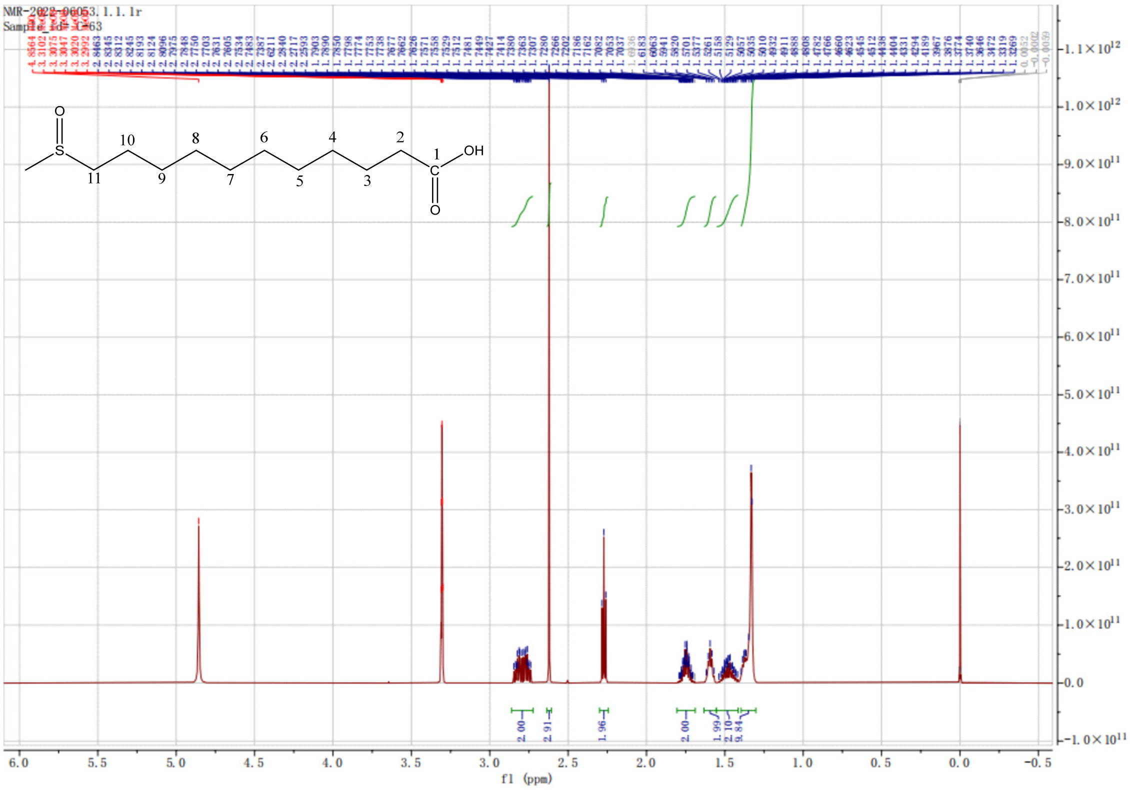

Supplementary Figure S4. ^{1}H-NMR spectrum of **2**

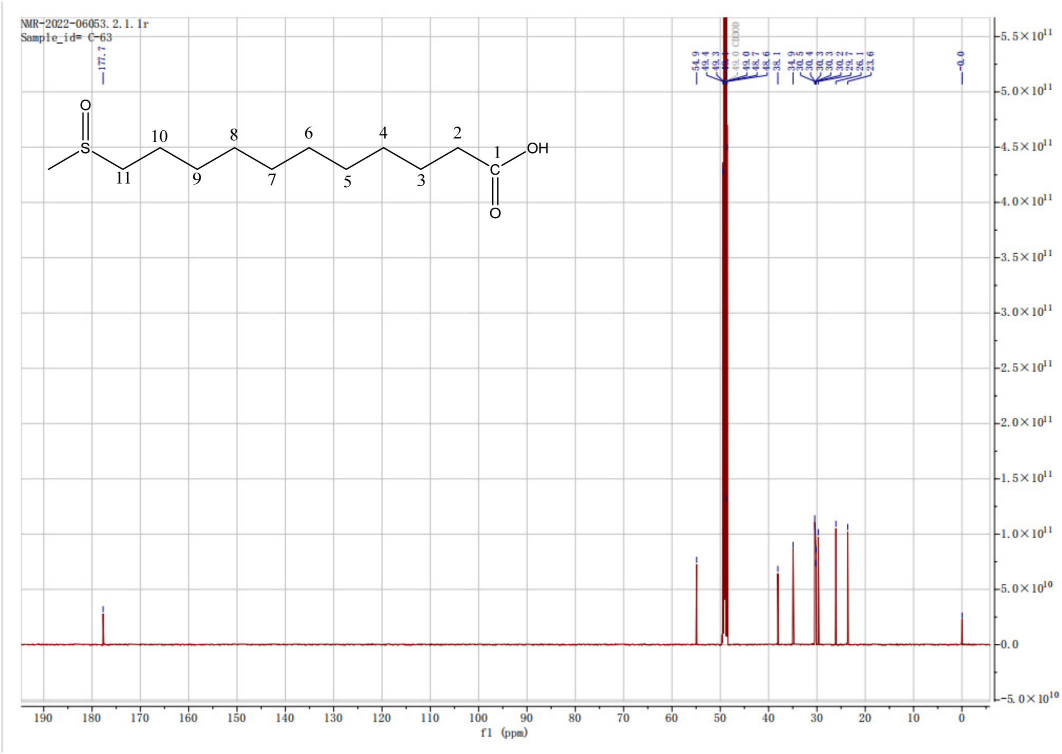

Supplementary Figure S5. ^{13}C-NMR spectrum of **2**

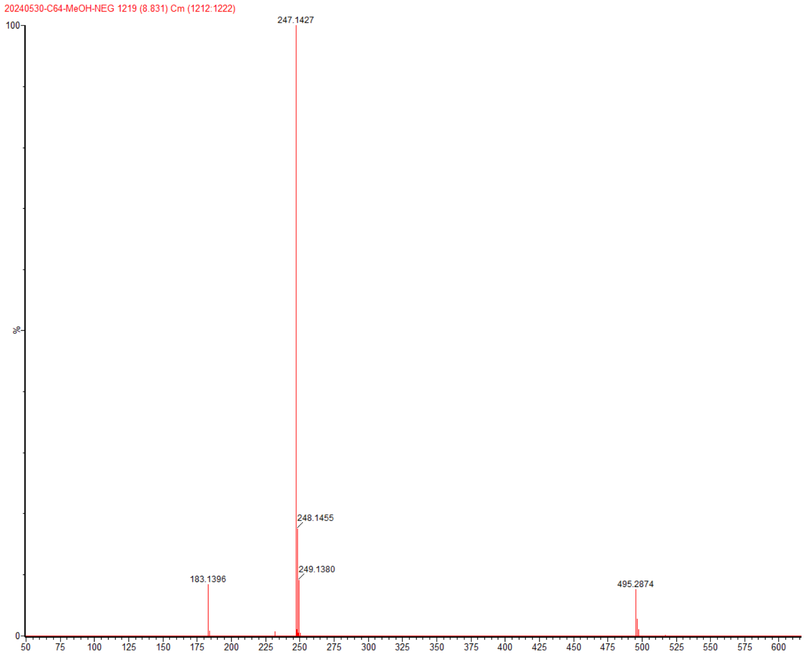

Supplementary Figure S6. HR-ESI-MS spectrum of **2**

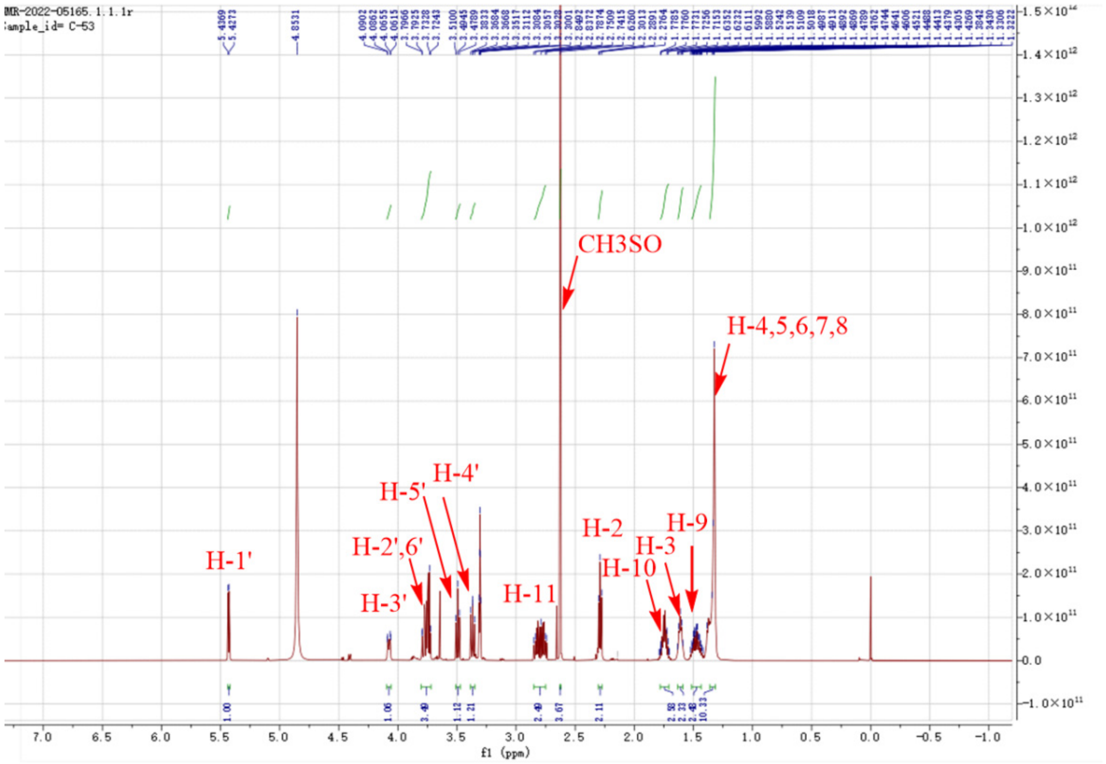

Supplementary Figure S7. ^{1}H-NMR spectrum of **3**

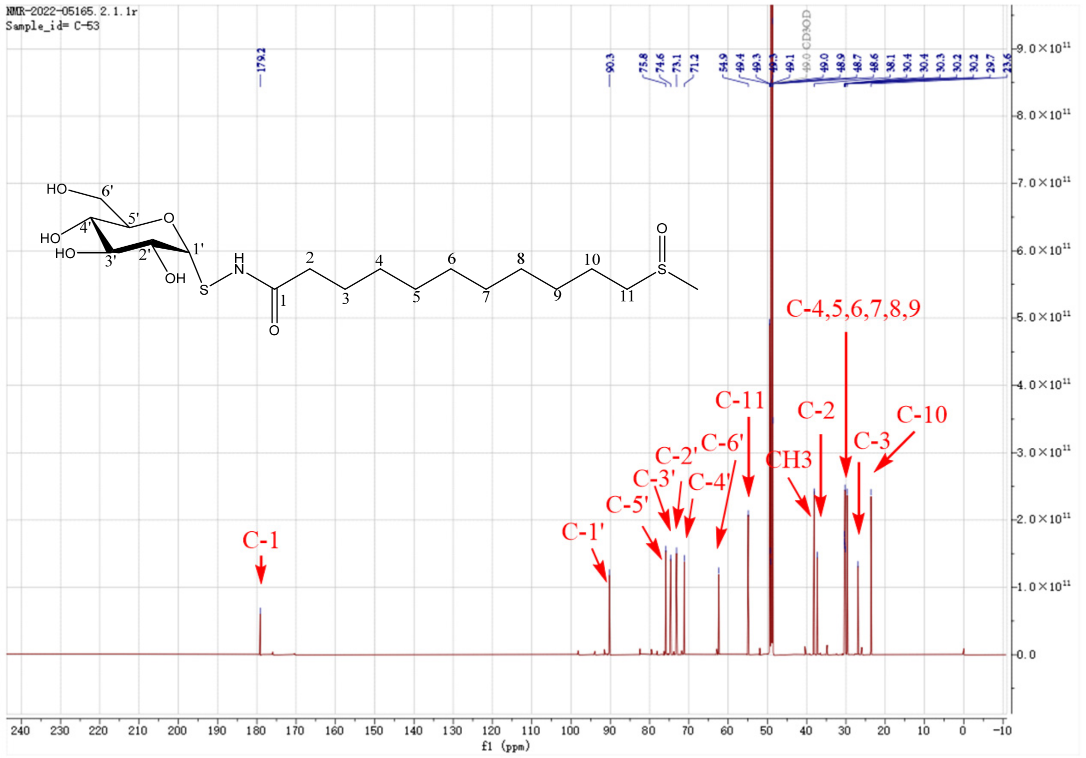

Supplementary Figure S8. ^{13}C-NMR spectrum of **3**

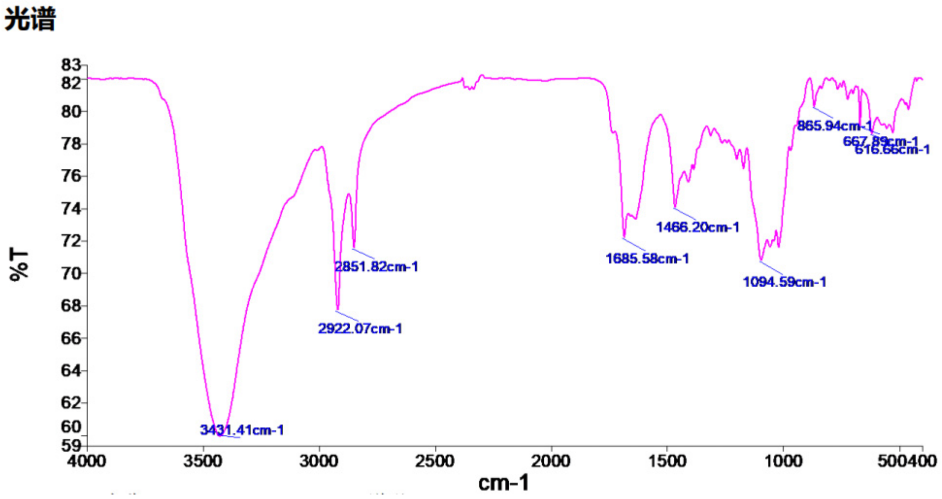

Supplementary Figure S9. IR spectrum of **3**

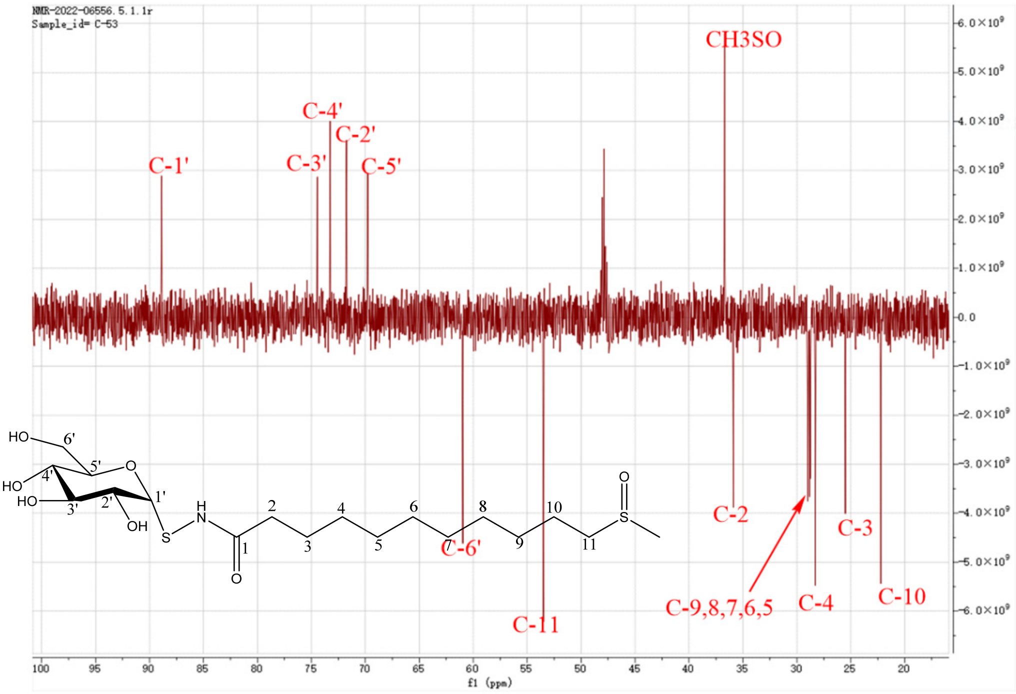

Supplementary Figure S10. DEPT spectrum of **3**

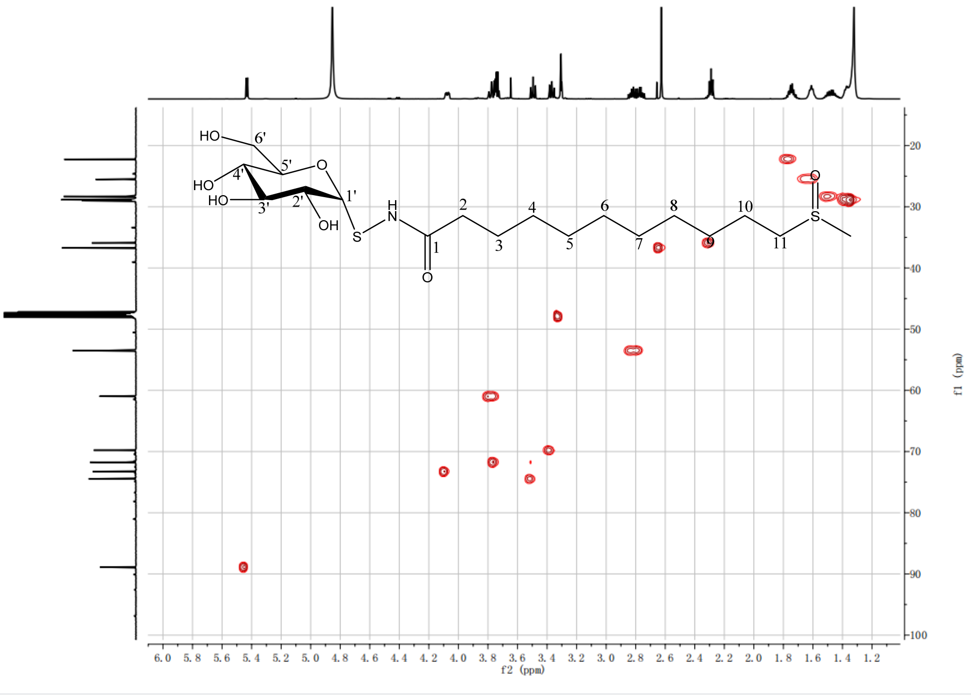

Supplementary Figure S11. HSQC spectrum of **3**

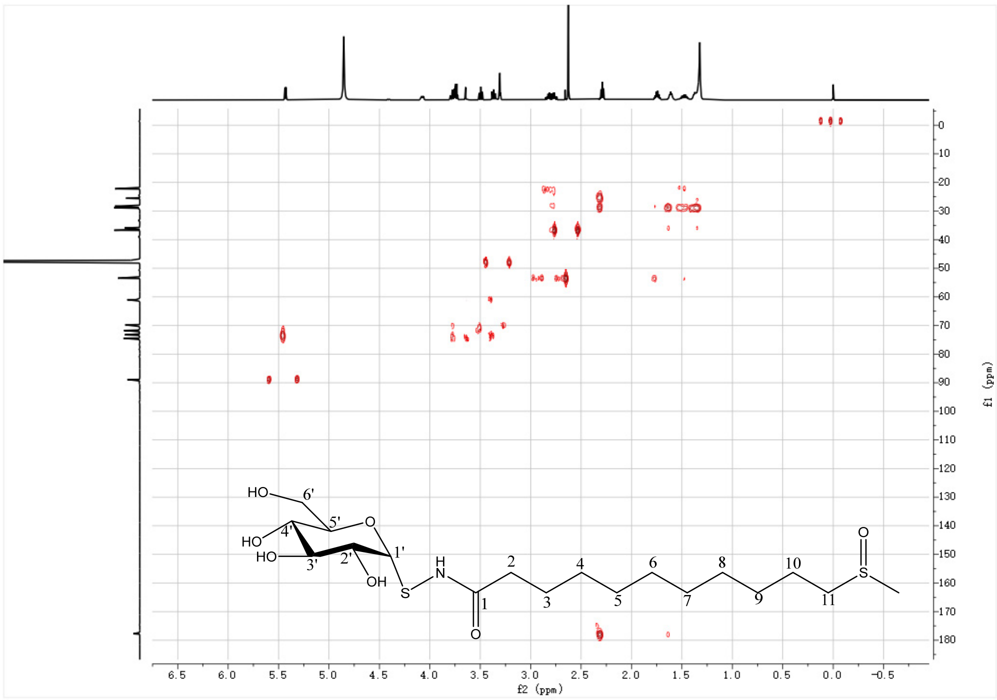

Supplementary Figure S12. HMBC spectrum of **3**

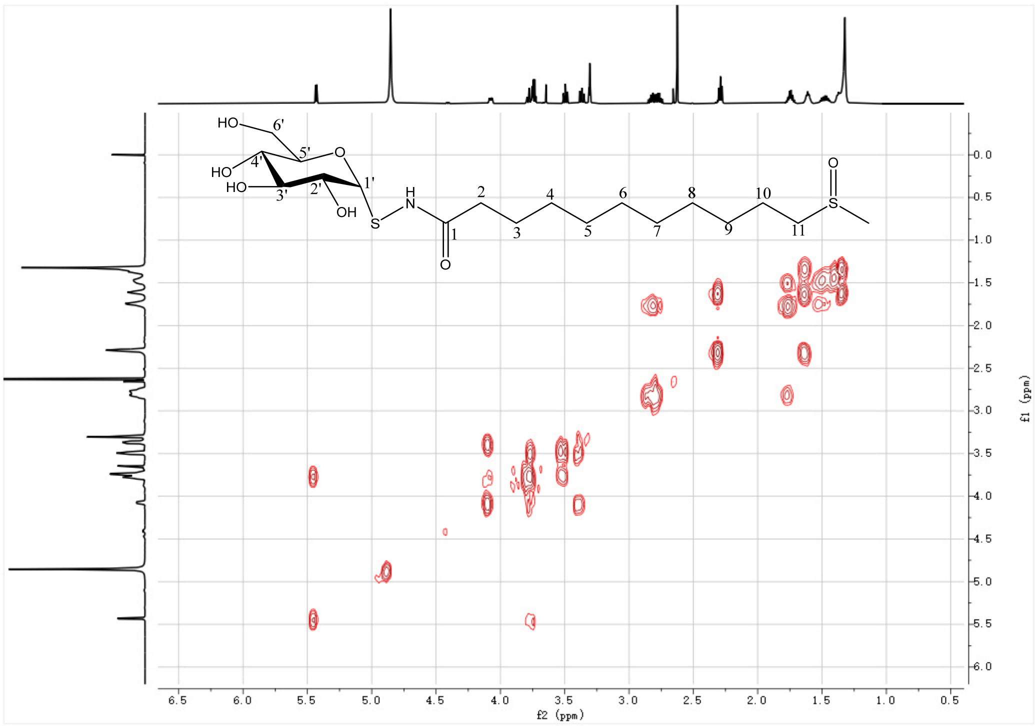

Supplementary Figure S13. ^{1}H-^{1}H COSY spectrum of **3**

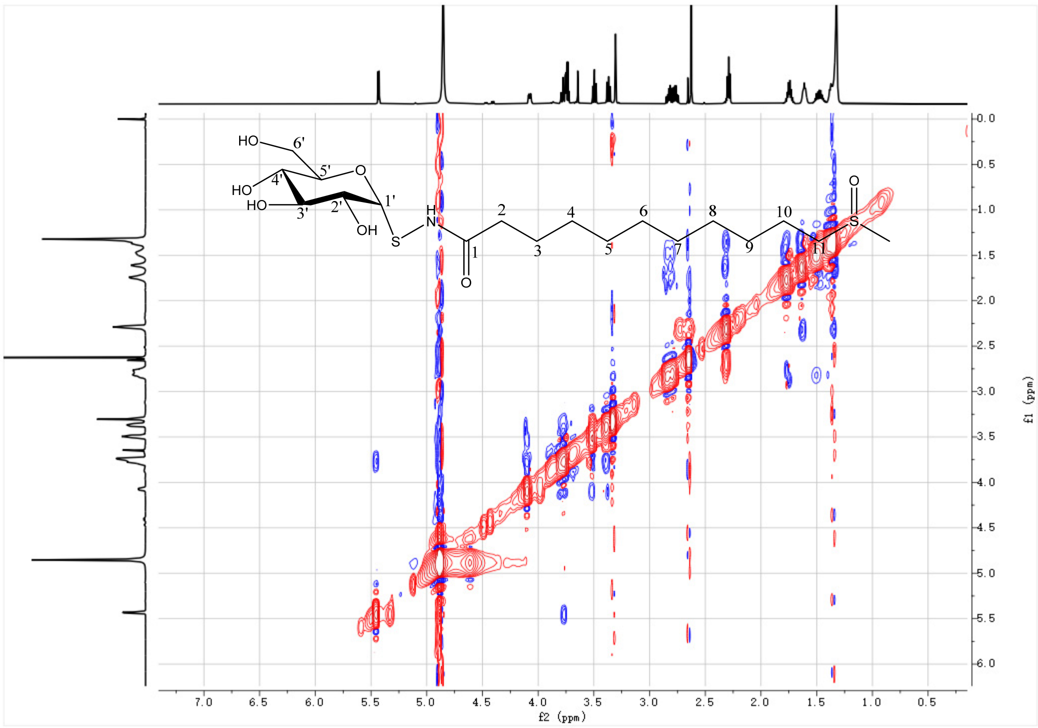

Supplementary Figure S14. NOESY spectrum of **3**

Qualitative Analysis Report

**Data Filename**	5723.d	**Sample Name**	C-53
**Instrument Name**	TOF G6230A	**Acquired Time**	2022-10-21
**Acq Method**	YCL.M	**Acquired SW**	6200 series TOF/6500 series
**IRM Calibration Status**	**Success**		
**User Chormatograms**			

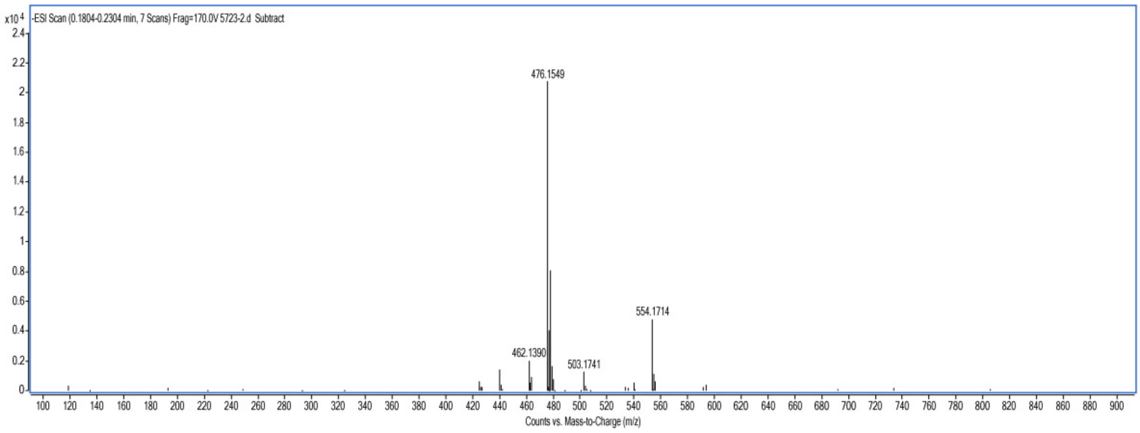

Supplementary Figure S15. HR-ESI-MS spectrum of **3**

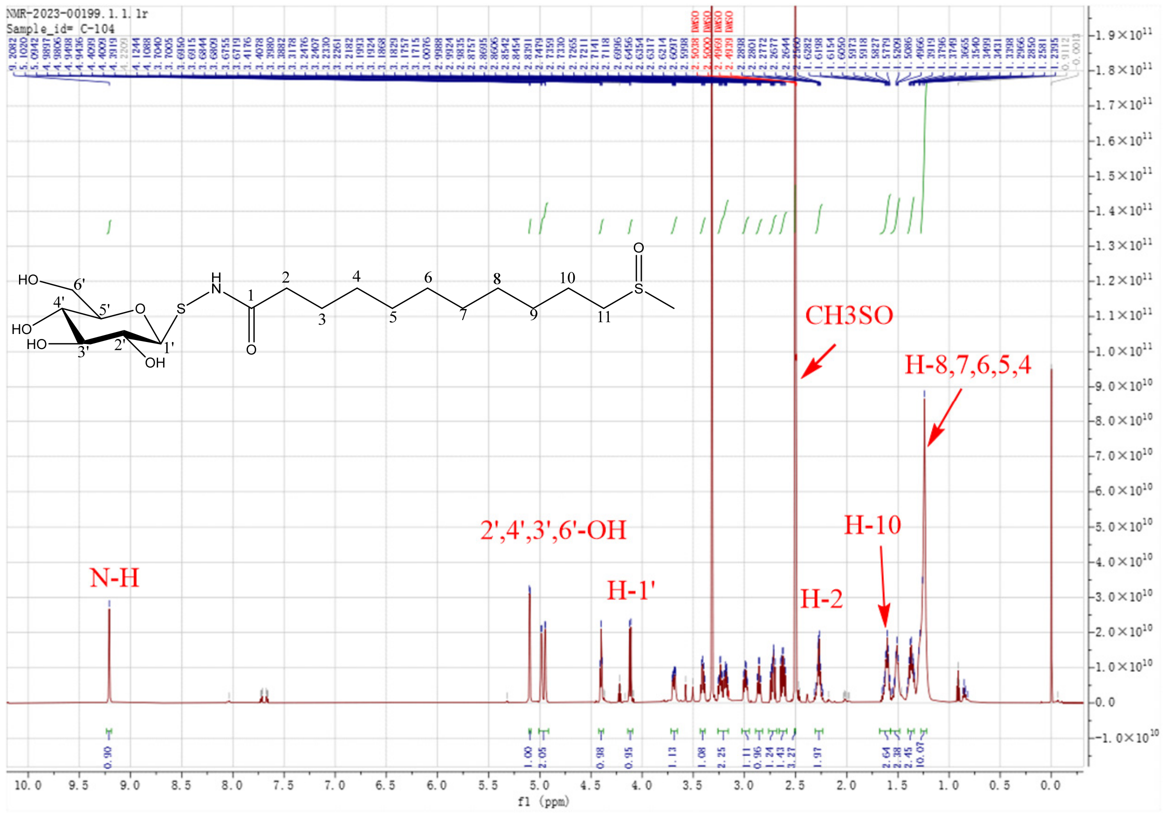

Supplementary Figure S16. ^{1}H-NMR spectrum of **4**

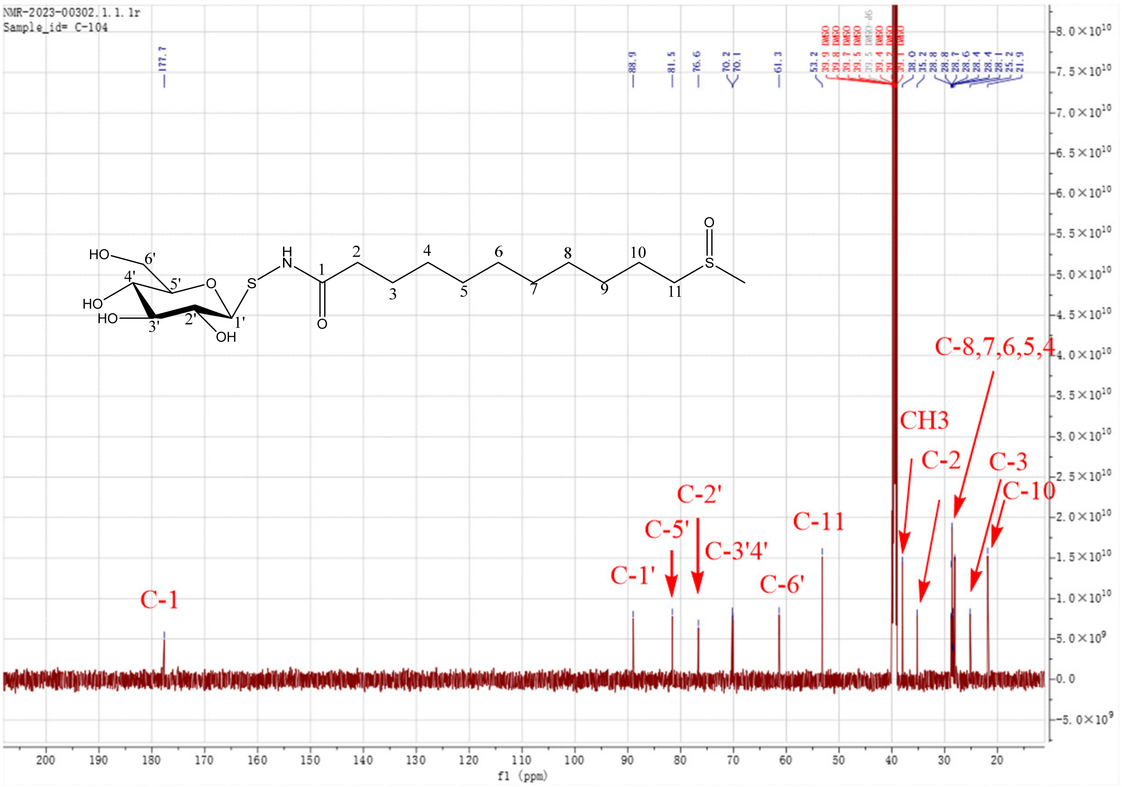

Supplementary Figure S17. ^{13}C-NMR spectrum of **4**

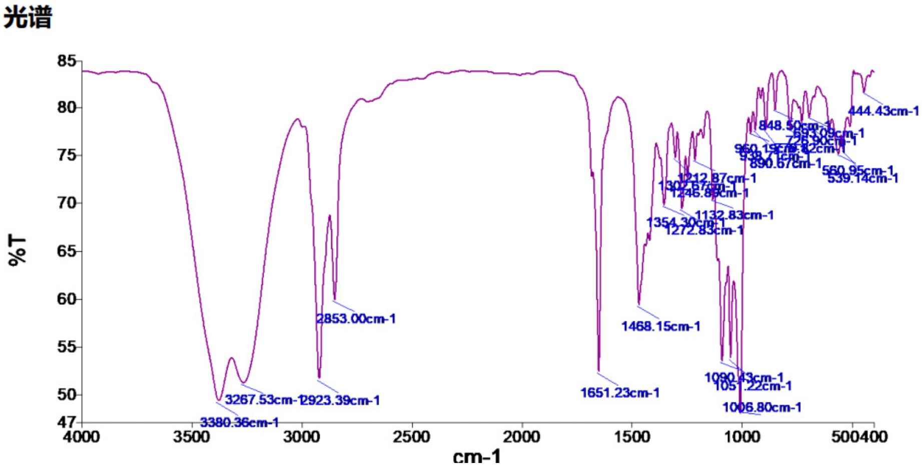

Supplementary Figure S18. IR spectrum of **4**

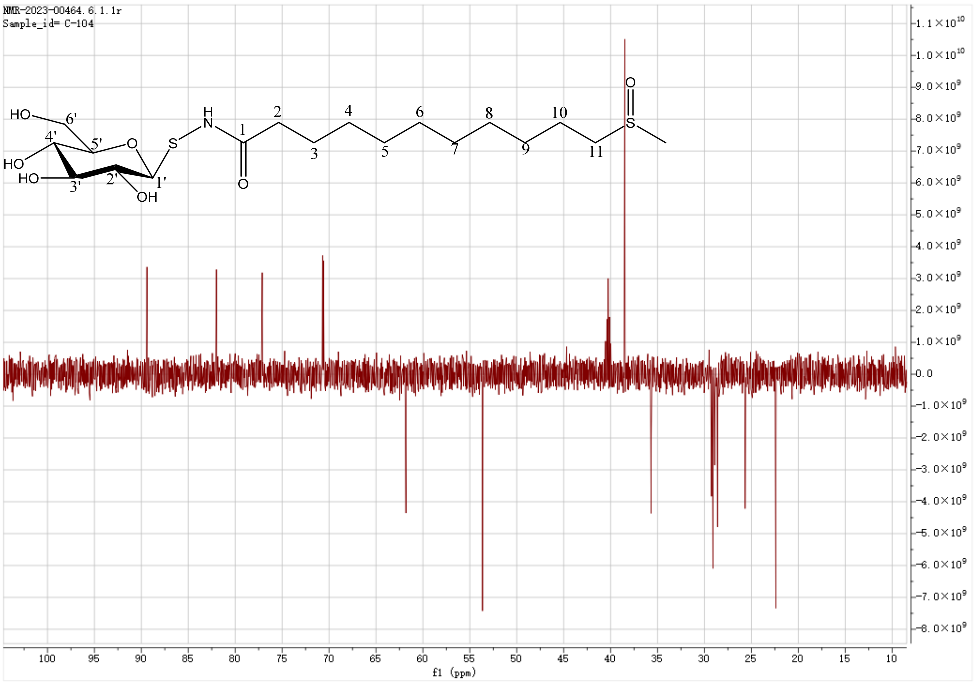

Supplementary Figure S19. DEPT spectrum of **4** (From top down: DEPT 135°, Original)

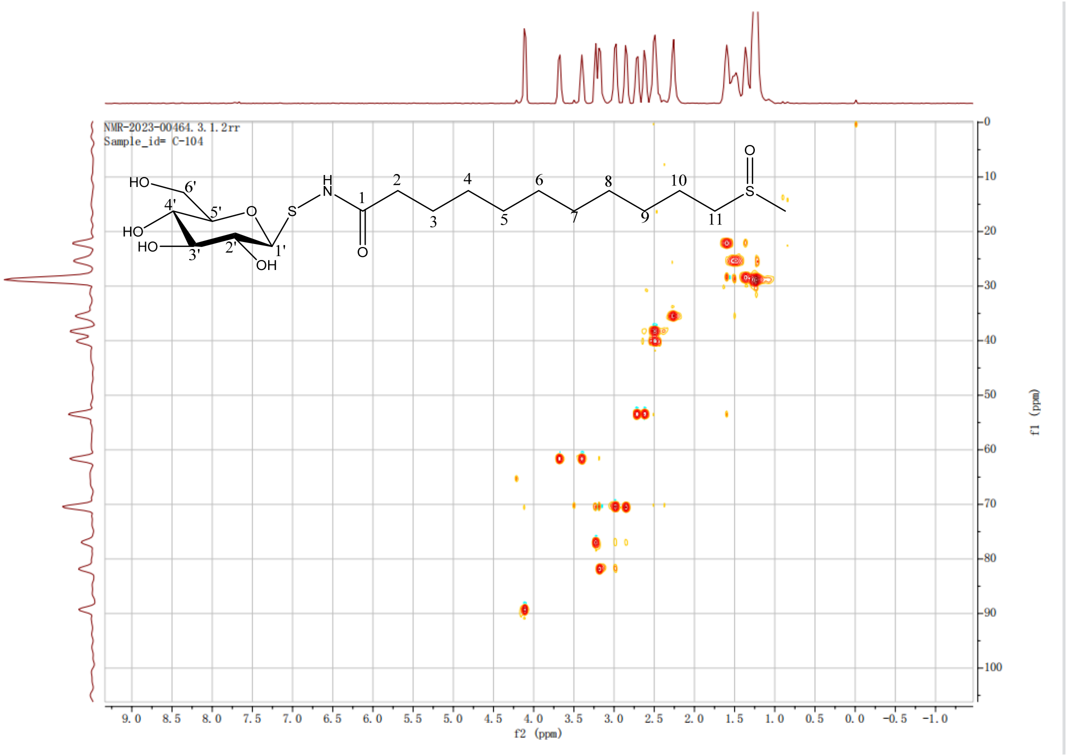

Supplementary Figure S20. HSQC spectrum of **4**

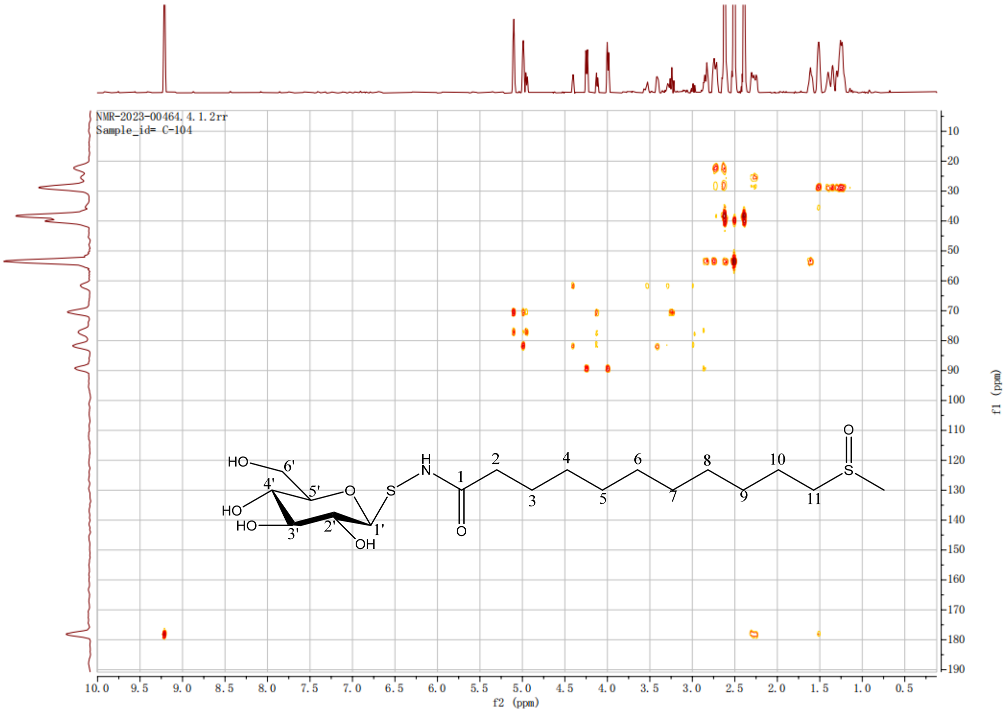

Supplementary Figure S21. HMBC spectrum of **4**

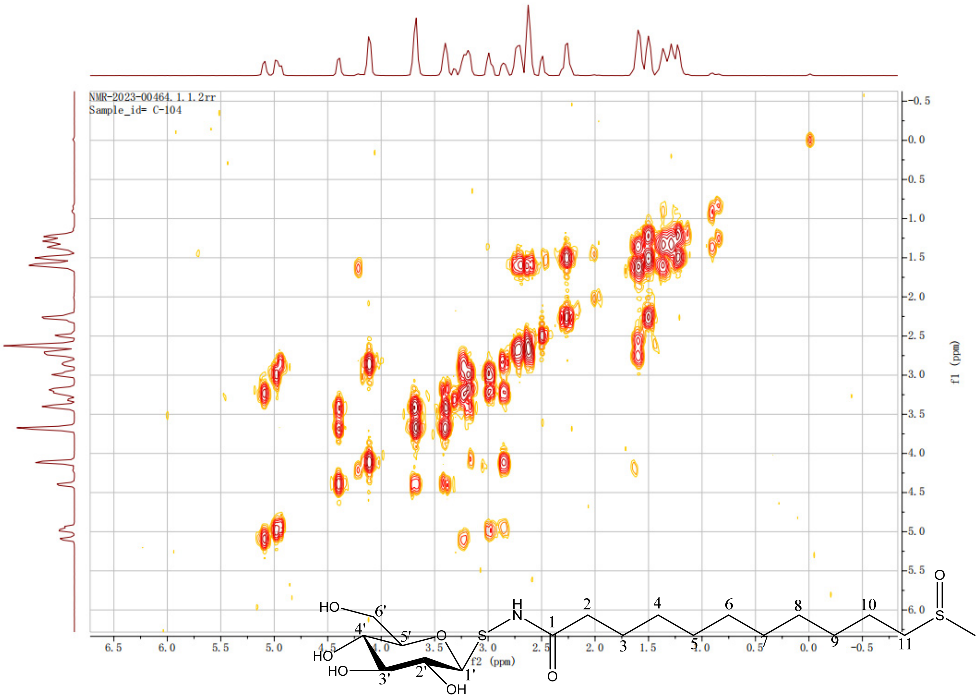

Supplementary Figure S22. ^{1}H-^{1}H COSY spectrum of **4**

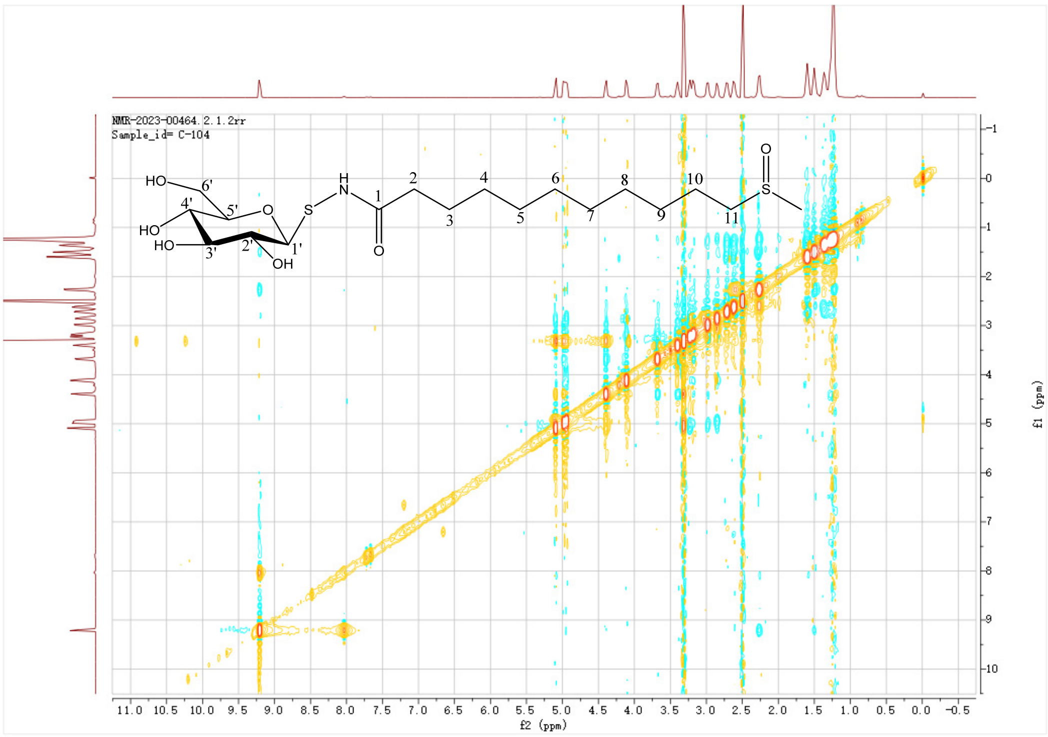

Supplementary Figure S23. NOESY spectrum of **4**

Qualitative Analysis Report

**Data Filename**	3117.d	**Sample Name**	W-104
**Instrument Name**	TOF G6230A	**Acquired Time**	2023-05-16
**Acq Method**	YCL.M	**Acquired SW**	6200 series TOF/6500 series
**IRM Calibration Status**	Success		
**User Chormatograms**			

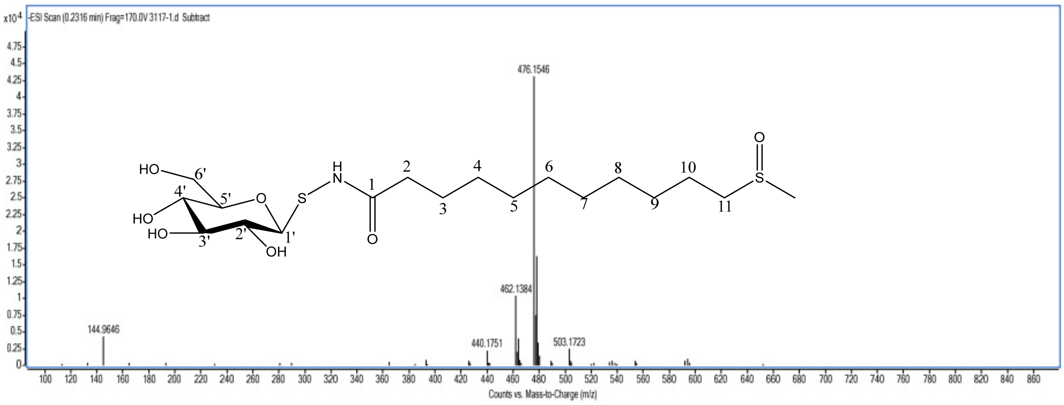

Supplementary Figure S24. HR-ESI-MS spectrum of **4**

Supplementary Table S1 NMR data of **1** and **2** (600 MHz for ^{1}H; 150 MHz for ^{13}C)

No	Compound **1**		Compound **2**	
	δ_H (*J* in Hz)	δ_C	δ_H (*J* in Hz)	δ_C
1	-	174.6	-	177.7
2	2.19, t(7.4)	33.7	2.27, t(7.4)	34.9
3	1.61, m	22.0	1.75, m	26.1
4	1.25-1.30, m	28.6	1.33-1.40, m	29.7
5	1.25-1.30, m	28.6	1.33-1.40, m	30.3
6	1.25-1.30, m	28.7	1.33-1.40, m	30.3
7	1.25-1.30, m	28.7	1.33-1.40, m	30.4
8	1.38, m	28.2	1.33-1.40, m	30.5
9	1.48, m	24.5	1.48, m	30.2
10	2.63,m; 2.72, m	53.2	1.59, m	23.6
11		-	2.79, m	54.9
CH_3SO	2.51, s	38.0	2.62, s	38.1
